# Regulatory Effect of Connexin 43 on Basal Ca^2+^ Signaling in Rat Ventricular Myocytes

**DOI:** 10.1371/journal.pone.0036165

**Published:** 2012-04-27

**Authors:** Chen Li, Qingli Meng, Xinfeng Yu, Xian Jing, Pingxiang Xu, Dali Luo

**Affiliations:** Department of Pharmacology, School of Chemical Biology & Pharmaceutical Sciences, Capital Medical University, Beijing, China; Istituto Dermopatico dell'Immacolata, Italy

## Abstract

**Background:**

It has been found that gap junction-associated intracellular Ca^2+^ [Ca^2+^]_i_ disturbance contributes to the arrhythmogenesis and hyperconstriction in diseased heart. However, whether functional gaps are also involved in the regulation of normal Ca^2+^ signaling, in particular the basal [Ca^2+^]_i_ activities, is unclear.

**Methods and Results:**

Global and local Ca^2+^ signaling and gap permeability were monitored in cultured neonatal rat ventricular myocytes (NRVMs) and freshly isolated mouse ventricular myocytes by Fluo4/AM and Lucifer yellow (LY), respectively. The results showed that inhibition of gap communication by heptanol, Gap 27 and flufenamic acid or interference of connexin 43 (Cx43) with siRNA led to a significant suppression of LY uptake and, importantly, attenuations of global Ca^2+^ transients and local Ca^2+^ sparks in monolayer NRVMs and Ca^2+^ sparks in adult ventricular myocytes. In contrast, overexpression of rat-Cx43 in NRVMs induced enhancements in the above measurements, and so did in HEK293 cells expressing rat Cx43. Additionally, membrane-permeable inositol 1,4,5-trisphosphate (IP_3_ butyryloxymethyl ester) and phenylephrine, an agonist of adrenergic receptor, could relieve the inhibited Ca^2+^ signal and LY uptake by gap uncouplers, whereas blockade of IP_3_ receptor with xestospongin C or 2-aminoethoxydiphenylborate mimicked the effects of gap inhibitors. More importantly, all these gap-associated effects on Ca^2+^ signaling were also found in single NRVMs that only have hemichannels instead of gap junctions. Further immunostaining/immunoblotting single myocytes with antibody against Cx43 demonstrated apparent increases in membrane labeling of Cx43 and non-junctional Cx43 in overexpressed cells, suggesting functional hemichannels exist and also contribute to the Ca^2+^ signaling regulation in cardiomyocytes.

**Conclusions:**

These data demonstrate that Cx43-associated gap coupling plays a role in the regulation of resting Ca^2+^ signaling in normal ventricular myocytes, in which IP_3_/IP_3_ receptor coupling is involved. This finding may provide a novel regulatory pathway for mediation of spontaneous global and local Ca^2+^ activities in cardiomyocytes.

## Introduction

In myocardium gap junctions provide both electrical and metabolic exchange among connected myocytes, enabling a synchronized excitation and muscle contraction. Hemichannels are precursors of gap junctions, assembled by six connexin subunits that span the lipid bilayer. Like conventional ion channels, hemichannels do not remain continuously open, instead, they flip between open and closed states regulated by multiple stimuli. For instances, reduction in extracellular Ca^2+^, membrane depolarization, mechanical stress, metabolic inhibition, low intracellular redox potential, activation of purinergic receptors and intracellular kinase activity have all been implicated in the activation of hemichannel [1]–[6].

It has been demonstrated that functional connexin hemichannels also exist in isolated ventricular myocytes [6]. Open hemichannels are nonselective conduits for small molecules and cations, allowing the release of ATP [1], [2], [7] and NAD^+^ [8], and the influx of Ca^2+^ and Na^+^ [9]. Upon pathological insults such as ischemia and oxidative stress, hemichannels and gap coupling have been found to allow the passage of small molecules that contribute to cell injury [10], [11].

Intracellular Ca^2+^ ([Ca^2+^]_i_) transient represents the global intracellular Ca^2+^ signaling, while Ca^2+^ sparks are the building blocks of intracellular Ca^2+^ activity that derive from local, rapid and transient Ca^2+^ release from a cluster of ryanodine receptor (RyR) activation in the sarcoplasmic reticulum [12]. Both of the signal modes are important in regulation of normal heart function. Previous studies have shown that under pathological condition gap coupling is disordered and involved in the abnormal Ca^2+^ activities that potentially generate lethal arrhythmias and hyperconstriction in ventricles [11], [13]–[16], suggesting a functional role of the gap junction/intercellular communication in the regulation of Ca^2+^ signaling in diseased heart. Yet whether gap junction and hemichannels are also involved in the modulation of Ca^2+^ signaling, particularly, in the basal Ca^2+^ activities in normal heart, is presently unknown.

In this study, we used single cardiac myocytes to determine the effects of hemichannel on the [Ca^2+^]_i_ activities and compared them with those found in monolayer myocytes that already form typical gap junctions. We found that both confluent and single myocytes exhibited downregulated Ca^2+^ signaling in response to gap uncouplers and interference of connexin43 (Cx43) expression the predominant connexin in the ventricles, while overexpression of Cx43 displayed enhanced Ca^2+^ activities in both densities of the cells. Therefore, this study demonstrates that Cx43-associated coupling plays a fundamental role in the mediation of local and global Ca^2+^ signaling in ventricular myocytes.

## Materials and Methods

### Materials and animals

Fluo-4/AM and Lucifer yellow (LY) were obtained from Molecular Probes (Invitrogen Inc, Carlsbad, California, USA). Myo-inositol 1,4,5-trisphosphate hexakis (butyryloxymethyl) ester (IP_3_/BM) was synthesized as instructed [17] (purity>95%). Xestospongin C was purchased from Calbiochem (Merck Inc, Darmstadt, Germany). All the antibodies and the reagents used, unless otherwise indicated, were purchased from Santa Cruz Biotechnology, Inc (Santa Cruz, CA. USA) and Sigma-Aldrich (St Louis, MO, USA), respectively.

C57BL mice (25–30 g) were obtained from the Experimental Animal Center of Capital Medical University (Beijing, China). The animals were housed at the animal care facility at 25°C with 12/12 h light/dark cycles and have free access to food and water ad libitum. All animal study protocols were approved by the Institutional Animal Research and Ethics Committee of Capital Medical University (Beijing, China, SCXK2009-0008).

### Isolation and culture of neonatal rat ventricular myocytes

NRVMs were isolated from 1 to 2-day-old Sprague-Dawley rats by enzymatic digestion with 0.1% trypsin and 0.03% collegenase, as described [18]. After removing cardiac fibroblasts, NRVMs were plated onto 60 mm or 35 mm dishes at a density of 1×10^6^ cells/ml for monolayer or dilute 10-fold for single cell study in Dulbecco's modified Eagle's medium containing 10% fetal bovine serum, 100 units/ml penicillin/streptomycin, and 0.1 mM 5-bromo-2-deoxyuridine to inhibit fibroblast proliferation.

### Isolation of ventricular myocytes from adult mice

C57BL mice were treated with heparin (2.5 units/g body weight) by intraperitoneal injection for 15 min before obtaining their hearts for perfusion. After proper anesthesia (10% chloral hydrate was intraperitoneally injected at 0.1 ml/20 g), the heart was rapidly excised and dropped into a beaker of cold modified HEPES buffered Tyrode solution (in mM NaCl 120, KCl 5.4, NaH_2_PO_4_ 1.2, MgSO_4_ 1.2 and glucose 5, HEPES 5, tuarine 5, 2,3-butanedione monoxime 10). After perfusion with Ca^2+^-free Tyrode solution and equilibration with 95%O_2_∶5%CO_2_ in Langendorff preparation, the heart was incubated in the enzyme solution (0.5 mg/ml collegenase and 0.1 mg/ml trypsin) gassed at 37°C for 15 min. Then the ventricle was broken into pieces by forceps and titrated gently with aspiration pipette, and the dispersed cells were harvested and filtered by 200 micron-pore sized cell sieve. Cells were incubated with Medium 199 containing 10% calf serum before usage for 2 h.

### Confocal Ca^2+^ transient and spark imaging

Measurement of [Ca^2+^]_i_ and the preparation of HEPES buffered physiological saline solution (HBSS) were performed as previously described [18]. All the NRVMs were used after 48 h culture, while the adult mouse ventricular myocytes were immediately used after isolation and equilibration with M199. Experiments were performed at room temperature (22–24°C).

### LY uptake assay

NRVMs were incubated in a Ca^2+^-containing HBSS with the presence of vehicle or the drug of interest e.g. heptanol, Gap 27 and flufenamic acid (FFA) for 1–2 min, 30 min and 5 min, respectively. After washing with Ca^2+^-free HBSS twice, the cells were incubated with 2.5% LY (containing 1 mM EGTA) for 5 min at room temperature, and then the dye uptake was observed by Leica SP5 fluorescence laser scanning confocal microscopy (excitation at 405 nm and emission detection at 530 nm). Ten pictures were taken randomly from each dish for the statistical analysis.

### Cell infection with adenovirus

All the construction of plasmids and adenovirus were performed by Invitrogen Inc. (Shanghai, China). In brief, Cx43 coding region was amplified by PCR reaction. The sequences for the pair of primers are 5′- CCGCTCGAGGCCACCATGGGTGACTGGAGTGCCTTGGG-3′ and 5′-CCGGAATTCTTAAATCTCCAGGTCATCAGGCCGA-3′. Then the PCR products were digested by *XhoI* and *EcoRI* and cloned into pIRES2-EGFP vector. The Cx43 overexpressing plasmids pGJA1-IRES-EGFP (wt-Cx43) was confirmed by sequencing and subcloned into pAD/CMV/V5-DEST vector by gateway reconstitution technique to make pAd-JX-GJA1-IRES2-EGFP adenovirus construct. To make the Cx43 knockdown plasmids, the complementary sequences 5′-TGCTGGATTCGCGTCTTCTTGTTGTCGTTTTGGCCACTGACTGACGACAACAAAGACGCGAATC-3′ and 5′-CCTGGATTCGCGTCTTTGTTGTCGTCAGTCAGTGGCCAAAACGACAACAAGAAGACGCGAATCC-3′ were verified to avoid the off-target silencing and inserted into pcDNA™6.2-GW/EmGFPmiRNA vector using BLOCK-iT™ Pol II miR RNAi Expression Vector Kit. After evaluation of the knockdown effects, it was also subcloned into pAD/CMV/V5-DEST vector. NRVMs were infected with adenovirus constructs (m.o.i. = 15) for 48 h to examine the overexpression or knockdown of Cx43 protein. Additionally, HEK293 cells were also expressed with wt-Cx43 by plasmid transfection (2 µg/ml) as previously described [19].

### Immunocytochemistry

Cardiac myocytes were fixed in 4% formaldehyde in phosphate-buffered saline (PBS) for 10 min and then permeabilized with 1% Triton X-100 PBS for 8 min at room temperature. After blocking in PBS containing 5% bovine serum albumin for 1 h, anti-Cx43 antibody was used overnight at 4°C at a dilution of 1∶100. The secondary antibody Alexa Fluor 488-labeled donkey anti-rabbit (Molecular Probes, Carlsbad, California, USA) were applied at a dilution of 1∶500 for 1 h at room temperature. The nucleus was labeled with Hoechst 33258 (1 µg/ml) for 5 min. Chemifluorescent detection was performed directly on a laser-scanning confocal microscopy (Leica SP 5) with a ×63 oil-immersion objective (NA 1.4). All negative controls were performed by taking host serum as the primary antibody.

### Western blot

The extraction of non-junctional and junctional protein lysates was performed as previously described with some modification [20], [21]. In brief, NRVMs were incubated in lysis buffer (25 mM Tris-HCl, 150 mM NaCl, 2 mM EDTA, 2 mM EGTA, 1% Triton X-100) supplemented with 1 mM polymethylsulfonyl fluoride and 1× complete protease inhibitor cocktail on ice for 30 min. These samples were separated into Triton-soluble and -insoluble fractions by centrifugation at 14,000 rpm at 4°C for 30 min. Triton-insoluble fractions (pellets) were resuspended in the above lysis buffer supplemented with 2% Triton X-100 and 0.4% SDS and followed by brief sonication. After incubation on ice for 30 min, the lysates were centrifuged at 14,000 rpm at 4°C for 30 min to get the junctional protein lysates.

For the whole cell lysate, NRVMs were lysed in RIPA buffer containing 1 mM polymethylsulfonyl fluoride and 1× complete protease inhibitor cocktail. Lysates were boiled for 5 min, resolved on a 10% SDS-PAGE gel and transferred to PVDF membrane. Membranes were blocked with 5% nonfat milk powder in Tris-buffered saline containing 0.1% (v/v) Tween 20 for 60 min at room temperature. Anti- Cx43, -GAPDH and -α-actin antibodies were used overnight at 4°C at dilution of 1∶1500, 1∶3000 and 1∶1500, respectively. The immunoblotted membrane was then incubated with horseradish peroxidase-conjugated secondary antibody for 1 h and immunoreactive bands were detected by using enhanced chemiluminescence.

### Statistics

Data were analyzed and presented as means ± (S.E.) of *n* measurements. When appropriate, statistical comparisons between groups were carried out with 2-way paired or unpaired Student's *t* test. The accepted level of significance was *P*<0.05.

## Results

### Impairment of dye uptake by gap junction inhibitors in NRVMs

**Figure 1:**
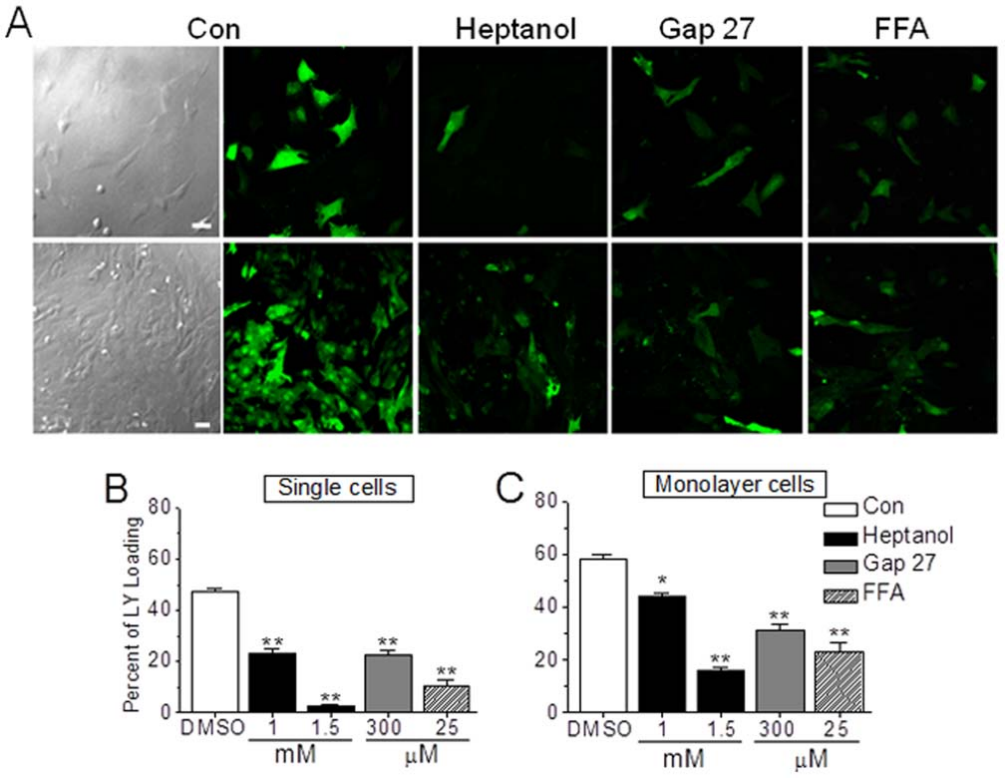
Impairment of dye uptake by gap junction inhibitors in NRVMs. (A) Typical confocal images of Lucifer yellow (LY) uptake in different groups of single and confluent NRVMs treated with DMSO or heptanol (1 or 1.5 mM) for 2 min, Gap 27 (300 µM) for 30 min and FFA (25 µM) for 5 min as indicated. (B, C) Statistical data were obtained from 6–8 independent determinations for each bar. * and ** represent *P*<0.05 and *P*<0.01, *vs.* DMSO, respectively.

To determine whether hemichannels in cardiac myocytes are functional, first we examined and compared the cellular uptake of LY in Ca^2+^-free extracellular medium between single and monolayer NRVMs. LY (molecular weight 457 Dalton) can only enter cells through gaps, thus providing a rapid and noninvasive approach to determine the activity of hemichannels by evaluation of dye spreading [22]. A spectrum of drugs has been shown to inhibit gap junction communication with variable degrees of efficacy and specificity. These drugs include heptanol (nonspecific) [23], Gap 27 (specific), a peptide that mimics short sequences in the extracellular loop 2 of Cx43 and inhibits gap junction by direct interaction with exposed hemichannels in plasma membranes [24], and FFA as a hemichannel blocker [25], [26]. As shown in Figure 1, inhibition of gap junction by 1 mM and 1.5 mM heptanol significantly reduced the dye uptake by 52.3±2.33% and 95.1±4.54% in single cells and 23.6±1.25% and 74.4±3.31% in monolayer cells. Also, Gap 27 (300 µM) and FFA (25 µM) attenuated the LY uptake by 52.4±2.32% and 46.5±1.19%, and 78.3±3.63% and 60.3±2.32% in single and monolayer cells, respectively. Thus, these data suggest that functional gaps present not only in confluent cardiomyocytes, but also in single cells, in this case as hemichannels.

### Inhibition of spontaneous Ca^2+^ signals by gap junction inhibitors in NRVMs

**Figure 2:**
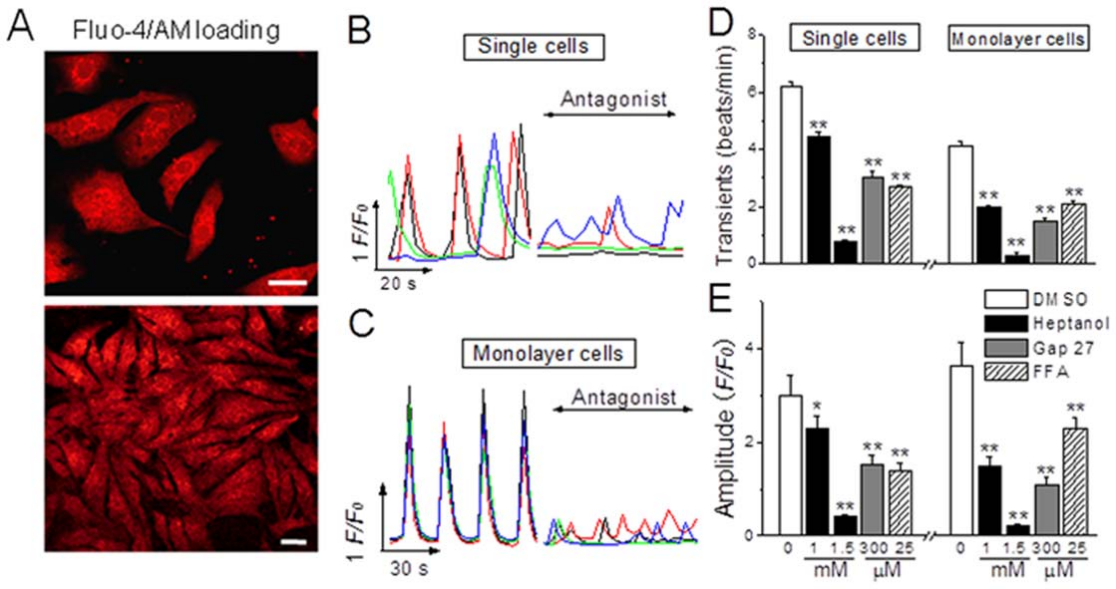
Inhibition of spontaneous Ca^2+^ signals by gap junction inhibitors in NRVMs. (A) The intracellular Ca^2+^ alterations in NRVMs, loaded with Fluo4/AM, were monitored by confocal microscopy. (B, C) Typical traces represent the spontaneous Ca^2+^ transients in single and monolayer NRVMs prior to and after heptanol (1 mM or 1.5 mM) for 2 min, Gap 27 (300 µM) for 30 min or FFA (25 µM) for 5 min. (D, E) The statistical data of the Ca^2+^ transient frequency and amplitude in single and confluent NRVMs as indicated were obtained from 10–12 independent determinations for each bar. * and ** represents *P*<0.05 and *P*<0.01 *vs.* DMSO, respectively.

To determine if gap junctions regulate the resting Ca^2+^ signaling in ventricular myocytes, we monitored the spontaneous Ca^2+^ signals in unstimulated confluent NRVMs or non-contacting single NRVMs with or without the presence of gap uncouplers. As shown in Figure 2, both single and confluent NRVMs oscillated spontaneously at different rhythm, and it is obvious that monolayer cells displayed coordinate Ca^2+^ oscillations because of the formation of gap junctions (Figure 2A and C), whereas single cells generated uncoupled Ca^2+^ transients among the sighted cells due to lack of gap junctions (Figure 2A and B). Heptanol at the similar concentrations used for LY measurement could significantly inhibit the frequency and amplitude of the spontaneous Ca^2+^ transients in both single and monolayer cells, whereas the duration of the transient remained unaltered (Figure 2D and E). Similarly, Gap 27 and FFA also remarkably hindered the Ca^2+^ transients by reducing transient frequency and amplitude in either density of NRVMs, respectively. It appeared that all the gap uncouplers attenuated the Ca^2+^ transients with more potency in confluent cells than in single cells, likely implying that the hemichannels in single cells were not so sensitive to uncoupler as the hemichannels/gap channels in confluent cells. Nevertheless, these observations do demonstrate an obvious effect of hemichannel/gap channels on the resting global Ca^2+^ signaling in ventricular myocytes.

### Role of Cx43 in global Ca^2+^ signal in NRVMs and non-muscle cells

**Figure 3:**
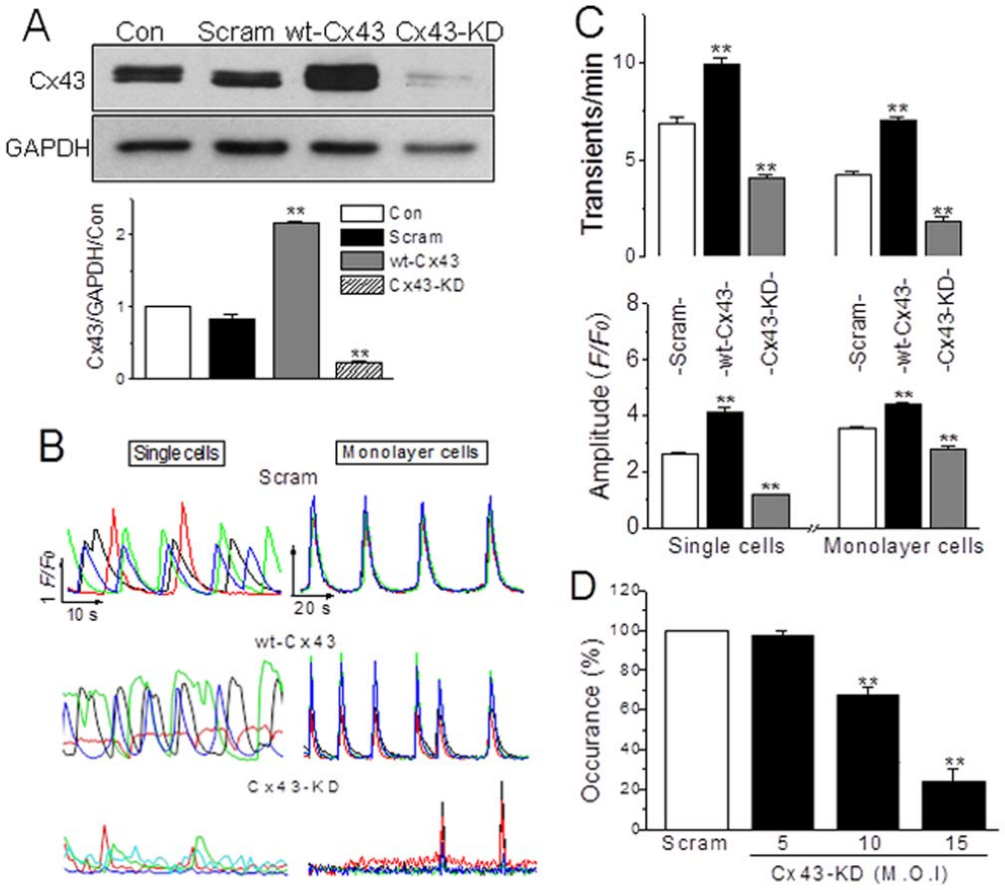
Role of Cx43 in gap-mediated Ca^2+^ transients in NRVMs. (A) NRVMs were infected with adenovirus vector or adenovirus carrying Cx43 full sequences (wt-Cx43) or Cx43 siRNA (m.o.i. = 15) for 48 h. The expression of Cx43 in NRVMs was determined by Western blot, which was normalized by the level of GAPDH. ** denotes *P*<0.01 vs. control. (B) Typical traces represent spontaneous Ca^2+^ transients in single and monolayer NRVMs with Cx43 overexpression or knockdown as indicated, and (C) Their statistical data of the transient frequency and amplitude were obtained from 5–6 independent experiments. (D) The statistical data of the dose-dependent effect of Cx43 deficiency on Ca^2+^ transients in single cells were obtained from 7–9 independent experiments. ** represents *P*<0.01 *vs.* scramble (Scram) for each panel.

The above results demonstrated that gap junction inhibitors impaired LY uptake as well as spontaneous [Ca^2+^]_i_ activity among monolayer NRVMs and in single NRVM. To assure if the gap permeability affected the Ca^2+^ signaling specifically, we constructed adenovirus carrying rat Cx43 gene with full sequences (wt-Cx43) and specific knockdown sequences encoding rat Cx43 (Cx43-KD, see Methods), the major connexin in ventricular myocytes. Transfection of the NRVMs for 48 h with wt-Cx43 or Cx43-KD virus displayed a significant overexpression or knockdown of Cx43 in these cells (Figure 3A).

**Figure 4:**
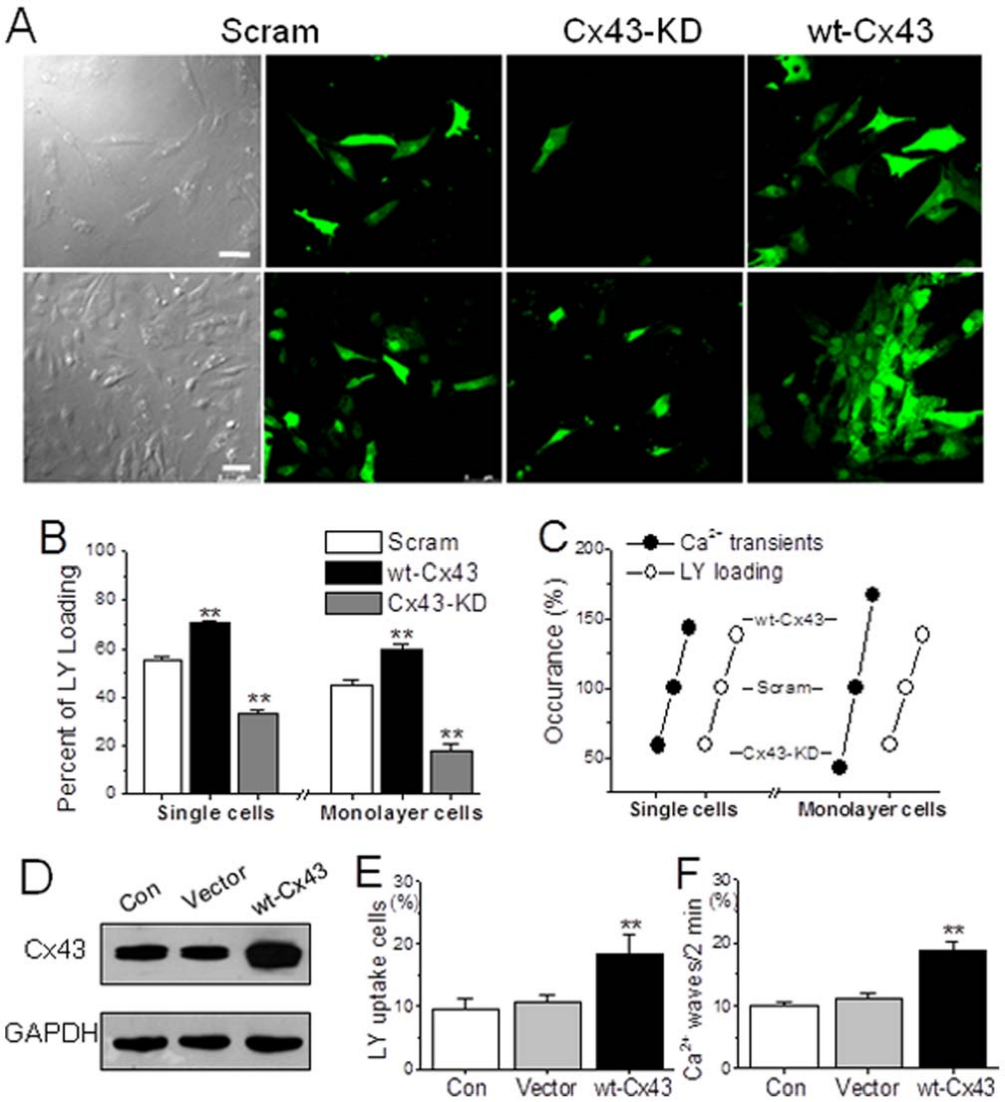
Role of Cx43 in LY uptake in NRVMs. (A, B) Typical confocal images of LY uptake in single and confluent NRVMs with Cx43 overexpression or silence by adenovirus infection (m.o.i. = 15). The LY uptake cells in each group as indicated were counted and expressed with percentage of the total recorded cells determined by 5–6 independent experiments for each bar. ** represents *P*<0.01 *vs.* scramble (Scram) cells. (C) Corresponding alterations of global Ca^2+^ transients and LY uptake in response to manipulations of Cx43 in both single and confluent monolayer NRVMs. (D) HEK293 cells were transfected with vector or plasmids carrying Cx43 for 48 h and determined their levels of Cx43 expression by Western blot. (E, F) Statistical data of the LY uptake percentage and Ca^2+^ wave frequency from different groups of HEK293 cells as indicated were obtained from 5–6 independent experiments. ** denotes *P*<0.01 *vs.* vector in all panels.

Then the effects of interfering Cx43 on spontaneous Ca^2+^ signals were examined in transfected NRVMs by the same protocol used above. In accordance with the findings in the uncoupler treatment, knockdown of Cx43 reduced the frequency and amplitude of Ca^2+^ transients, whereas overexpression of Cx43 greatly enhanced them in both single and monolayer NRVMs (Figure 3B and C). Furthermore, a Cx43 deficiency-dependent inhibitory effect on the Ca^2+^ oscillations was obtained by knocking down Cx43 with different concentrations of virus in single myocytes (Figure 3D). These data suggest a contribution of the endogenous Cx43-originated gaps/hemichannels for the spontaneous Ca^2+^ transient activity. At same time, corresponding results were also found in the evaluation of Cx43-associated permeability to LY, which was significantly blocked in Cx43-KD NRVMs, but dramatically potentiated in wt-Cx43 cells (Figure 4A and B). More importantly, taken the measurements in native NRVMs as control (100%), the values of LY transfer and Ca^2+^ transient rates were correlated well with the levels of Cx43 expression in both single and confluent transfected cells (Figure 4C).

To further confirm the Cx43 mediation of spontaneous Ca^2+^ signaling, HEK293 cells were adopted because these cells also possess endogenous Cx43 and can overexpress rat Cx43 by plasmid transfection. Additionally, this type of cells generate no-spreading spontaneous Ca^2+^ waves among connected cells that can be observed clearly under laser confocal microscopy, thus LY uptake and spontaneous Ca^2+^ waves were evaluated and compared between wt-Cx43 and vector-control cells. As shown in Figure 4D–F, rat Cx43 expression in HEK293 cells caused a similar effect as that found in muscle cells, i.e. significant increases in the evaluations of LY uptake and occurrence of Ca^2+^ waves.

### Role of Cx43 in local Ca^2+^ signal in cardiac myocytes

**Figure 5:**
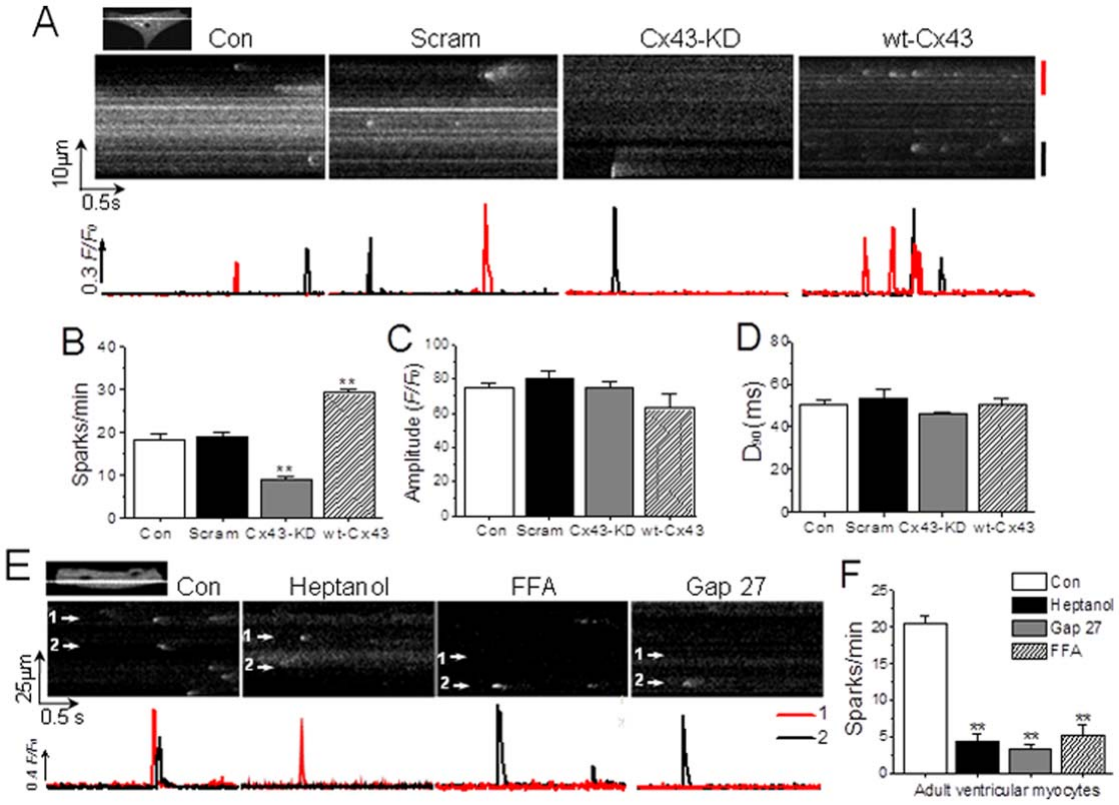
Effect of gap inhibition on Ca^2+^ sparks in NRVMs and adult mouse cardiomyocytes. (A) Typical linescan images of Ca^2+^ sparks (upper panel) and their *F/F_0_* changes over the time (lower panel) in single NRVM from different groups as indicated. (B, C, D) Statistical data of the spark frequency, amplitude and duration in NRVMs in different groups as indicated were obtained from 4–5 independent determinations, n = 20–36 cells for each panel. ** represents *P*<0.01 *vs.* scramble. (E) Typical linescan images of Ca^2+^ sparks (upper panel) and their *F/F_0_* changes over the time (lower panel) in adult mouse ventricular myocytes. The cells were respectively treated with vehicle (Con), 1.5 mM heptanol for 2 min, 300 µM Gap 27 for 30 min or 25 µM FFA for 5 min. (F) Statistical data from 3–5 independent determinations (n = 15–20 cells for each bar) show the effect of the drugs on the Ca^2+^ spark rate. ** represents *P*<0.01 *vs.* control.

Furthermore, the local Ca^2+^ signal like the sparks was examined too in resting NRVMs and freshly isolated adult mouse ventricular myocytes. Similar to the findings in global Ca^2+^ signal measurement, both gap junction inhibitors, heptanol and Gap 27 (data not shown), and interfering Cx43 expression in NRVMs significantly affected the frequency but not the amplitude and duration of spontaneous Ca^2+^ sparks in single and confluent NRVMs (Figure 5A–D). Similarly, the isolated adult ventricular myocytes also demonstrated decreased Ca^2+^ sparks due to gap inhibitors at the similar concentration used in neonatal myocytes (Figure 5E and F).

Therefore, both silencing and overexpressing Cx43 influenced the resting [Ca^2+^]_i_ activities and LY uptake in muscle and non-muscle cells, demonstrating a specific role of Cx43-associated coupling in the regulation of the fundamental Ca^2+^ signaling in these cells.

### Corresponding Cx43 alterations in transfected single and monolayer NRVMs

**Figure 6:**
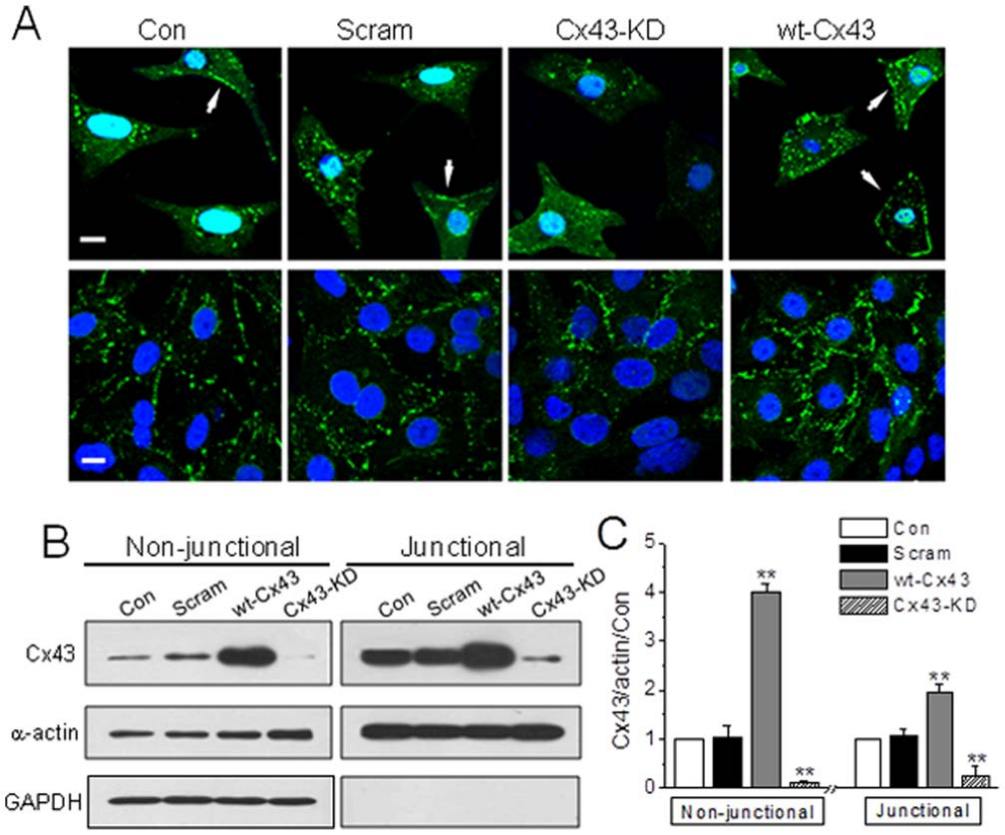
Distribution and expression level of Cx43 in single and monolayer NRVMs transfected with different virus. (A) Single and confluent NRVMs with Cx43 overexpression or knockdown by adenovirus infection (m.o.i. = 15) for 48 h. The subcellular distribution of Cx43 in different groups as indicated was determined by immunostaining the cells with antibody specific for Cx43. Cell nucleus are indicated by Hoechest 33258 (1 µg/ml), and the arrows indicate the membrane-associated Cx43. (B, C) NRVMs were infected with adenovirus vector or adenovirus carrying Cx43 full sequences (wt-Cx43) or Cx43 siRNA (Cx43-KD, both m.o.i. = 15) for 48 h. The expression of Cx43 in NRVMs was determined in Triton X-100 soluble and insoluble fractions by Western blot (B) and normalized by the level of α-actin and then the level of Cx43 in control cells (C). The detection of GAPDH in non-junctional but not in junctional fraction represents a successful separation of non-junctional and junctional Cx43. ** denotes *P*<0.01 *vs.* vector or scramble.

The interference of Cx43 by either gap inhibitors or gene manipulation demonstrated a significant disturbance in the basal Ca^2+^ signals in single NRVMs and adult cardiomyocytes, suggesting functional hemichannels possibly existed in these myocytes. Therefore, we examined the subcellular distribution of Cx43 in single cells and compared with that of monolayer cells by immunostaining and immunoblotting approaches. As shown in Figure 6A, typical punctuate Cx43 labeling was found in the interfaces between confluent NRVMs as reported, whereas the most Cx43 fluorescence was accumulated inside the cytosol in single NRVMs. However, membrane docked Cx43 indicated with white arrows in Figure 6A could be detected in some of the control and vector single cells (27.4±1.86% and 25.5±1.22%, n = 40 and 37 cells, respectively), and appeared to be increased in wt-Cx43 cells (34.2±1.43%, n = 35, *P*<0.05 *vs.* vector cells). The corresponding changes due to Cx43 overexpression and knockdown were further confirmed in western blotting Triton- insoluble and soluble fractions (see Methods), which represent gap junctions and precursors of gaps including hemichannels, respectively [20], [21]. While Cx43 was accordingly increased or reduced to Cx43-overexpression or Cx43-KD treatment in both soluble and insoluble fractions of monolayer cells (Figure 6B and C), Cx43 labeling was only detected in the soluble fraction of single cells that also responded to Cx43 manipulations (data not shown).

Therefore, the corresponding alterations in [Ca^2+^]_i_ activity to the level of non-junctional Cx43 observed in single cells suggest a role of functional hemichannel in the regulation of Ca^2+^ signaling in ventricular myocytes.

### Signaling pathways contributing to Cx43-mediated Ca^2+^ activities in NRVMs

**Figure 7:**
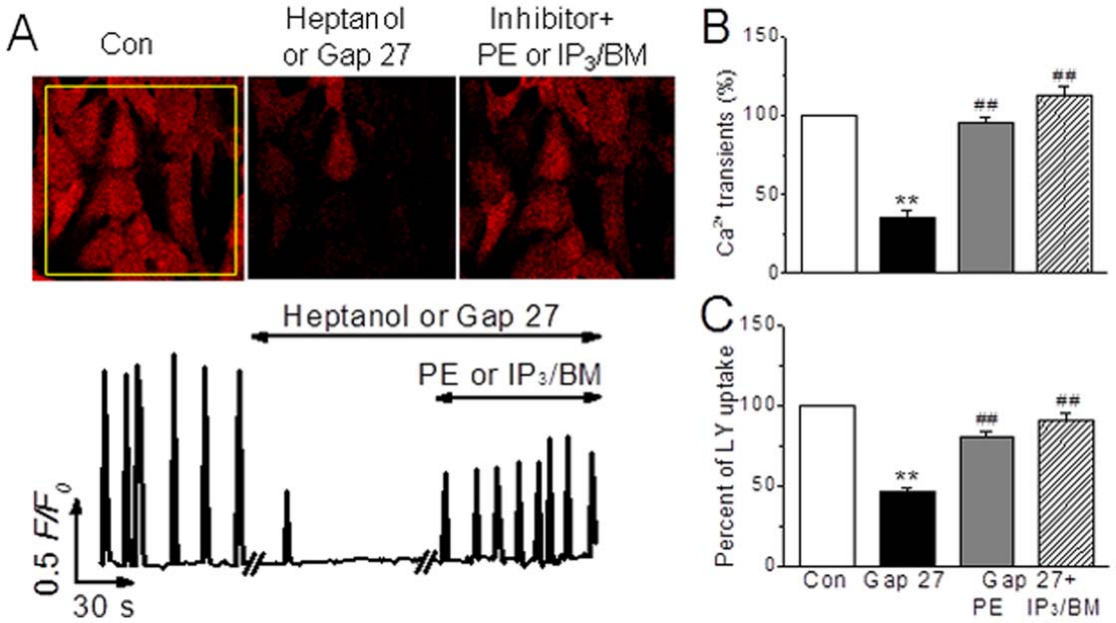
Recovery effect of inositol 1,4,5-trisphosphate on gap inhibitor-induced Ca^2+^ transient inhibition in monolayer NRVMs. (A) Images and traces represent spontaneous global Ca^2+^ oscillations in monolayer cells prior to and 1 or 30 min after heptanol (1 mM) or Gap 27 (300 µM), and then phenylephrine (PE 20 µM, 5 min) or IP_3_/BM (20 µM, 6 min) treatments. (B, C) The summarized data indicate the recovery effect of IP_3_ analogues on Gap 27-inhibited Ca^2+^ transients and LY uptake. **P<0.01 *vs.* control; ^##^ *P*<0.01 *vs.* Gap 27 alone, from 10–18 independent determinations for each bar.

**Figure 8:**
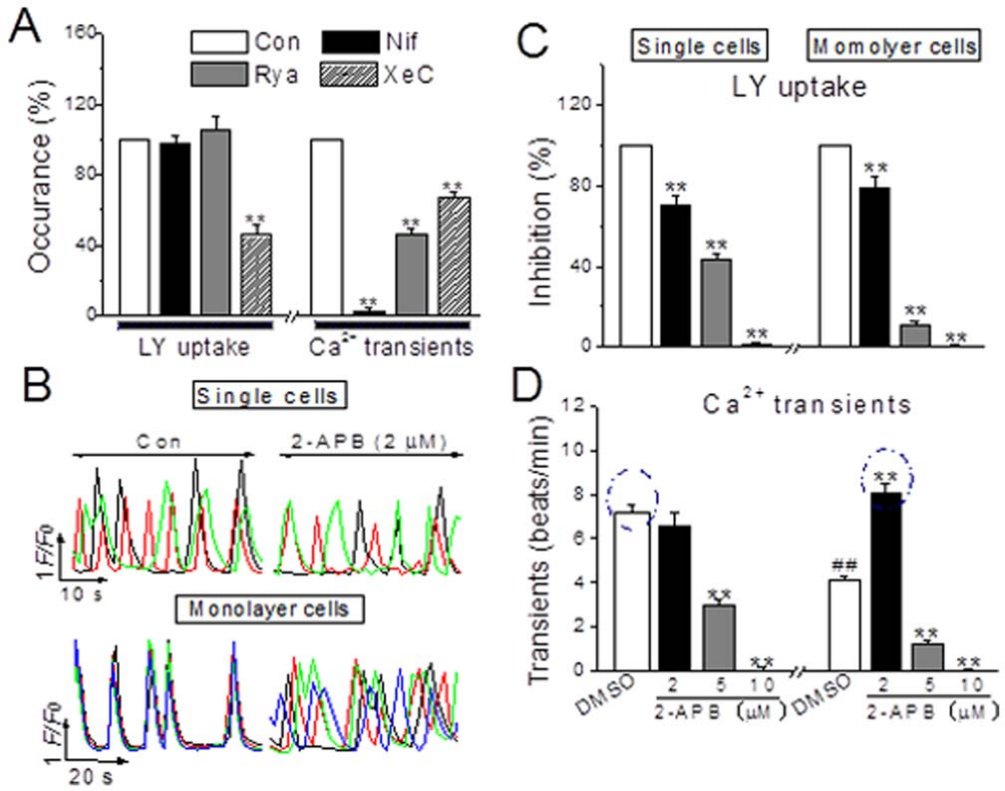
Pathways involved in the Cx43-associated mediation of Ca^2+^ activities in single and monolayer NRVMs. (A) The summarized data of the effects of nifedipine (Nif 1 µM, 10 min), ryanodine (Rya 100 µM, 10 min) and xestospongin C (XeC 10 µM, 20 min) on the LY uptake and intracellular Ca^2+^ frequency in NRVMs. (B) Typical traces represent the spontaneous Ca^2+^ transients in single and monolayer NRVMs prior to (Con) and after 2-APB (2 µM) for 10 min treatment. (C, D) The statistical data of the concentration-dependent effects of 2-APB on LY loading and Ca^2+^ transient frequency in single and confluent NRVMs as indicated were obtained from 8–12 determinations for each bar. **P<0.01 *vs.* DMSO in each panel; ^##^ *P*<0.01 *vs.* DMSO treated single cells.

To further investigate the mechanism(s) underlying the role of Cx43 in this regard, several important Ca^2+^ signaling pathways in the heart such as L-type Ca^2+^ channel/RyR and G-protein coupled receptors/inositol 1,4,5-trisphosphte receptor (IP_3_R) [12], [18], [27] and hemichannel-associated regulators like ATP and IP_3_ [7], [28]–[31] were examined. In monolayer NRVMs, phenylephrine (PE), an agonist of α-adrenergic receptor, and IP_3_/BM, a membrane permeable IP_3_, rescued the depressed occurrence of Ca^2+^ transients and LY loading induced by heptanol (1 mM) or Gap 27 (300 µM) (Figure 7A–C), but isoprenaline, an agonist of β-adrenergic receptor to activate L-type Ca^2+^ channels, and phorbol myristate acetate, an activator of PKC, showed no any effect (data not shown). Furthermore, like gap uncouplers, inhibition of IP_3_R with xestospongin C (XeC, 10 µM for 20 min), a selective IP_3_R inhibitor [18], caused significant inhibitions of LY loading and Ca^2+^ transients (Figure 8A). Due to an increase in basal [Ca^2+^]_i_ by XeC at higher concentration, another IP_3_R antagonist 2-aminoethoxydiphenyl borate (2-APB, 10 min) was used and it induced concentration-dependent blockades of LY uptake and Ca^2+^ transients in single and monolayer cells, except that 2-APB at concentration of 2 µM made the coordinate oscillations to desynchronized Ca^2+^ spikings in monolayer cells, mimicking the pattern of Ca^2+^ transients in single cells (Figure 8B–D). Additionally, the measurement of gap communication by fluorescence recovery after bleaching (FRAP) confirmed the above observation in LY loading (unpublished data). And, inhibitions of L-type channel with nifedipine (1 µM, 10 min) and RyR with ryanodine (100 µM, 10 min) induced tremendous suppression of Ca^2+^ oscillating, but the levels in LY uptake remained unchanged, a sign of normal gap communication, in these cells (Figure 8A), neither suramin, an antagonist of purinergic 2z/2x receptor, affected the gap inhibitor-regulated [Ca^2+^]_i_ activity and LY loading (data not shown). Thus, these results demonstrate IP_3_-IP_3_R pathway involves in the Cx43-mediated Ca^2+^ signaling, and further confirmations and study on signaling transduction are presented and submitted separately.

## Discussion

The present study demonstrated that functional Cx43-relevant gap junction/hemichannels play an important role in the regulation of spontaneous Ca^2+^ signaling in unstimulated NRVMs and adult myocytes. This proposal is mainly based on the following observations (i) in corresponding to the LY uptake inhibition, spontaneous Ca^2+^ transients and sparks were significantly affected by gap junction inhibitors and specific Cx43 interference in single and monolayer NRVMs; (ii) spontaneous Ca^2+^ sparks in adult cardiomyoctes displayed similar response to gap uncoupler treatments; (iii) overexpression of Cx43 in NRVMs or HEK293 cells exhibited enhancement in spontaneous Ca^2+^ activities and LY uptake; (iv) specific hemichannel inhibitor FFA showed a similar effect on [Ca^2+^]_i_ activities as gap uncouplers in neonatal and adult myocytes; and (v) like in monolayer cells, the changes in Ca^2+^ signals and LY uptake were well correlated with the level of cell surface Cx43 labeling and non-junctional Cx43 in single NRVMs.

In agreement with the chemical inhibitors of gap junction, specific overexpression or knockdown of Cx43 in NRVMs by adenovirus infection, the predominant connexin in ventricular myocytes, could upregulate or downregulate the gap permeability as well as the spontaneous Ca^2+^ signaling accordingly. In fact, a direct impact of Ca^2+^ spreading among adjacent cells has already been demonstrated in Cx43-expression manipulated cells in other studies. For instances, it has been shown that Cx43-expression in other cell types is correlated with both intercellular dye transfer [32], [33] and propagated intercellular Ca^2+^ waves through gaps [2], [34]. Similarly, mutation of Cx43 in NRVMs can induce desynchronization of Ca^2+^ transients that further hampers the synchronous beating [35]. All these studies demonstrate a critical role of gap junctions in the spreading of physiological Ca^2+^ signals among connected cells. The present study further depicts a role of Cx43-associated gap coupling in the regulation of intrinsic Ca^2+^ signaling inside the ventricular myocytes. Such effect of Cx43-coupling should be unrelated with the instant electrical exchange between myocytes, because similar observations found in monolayer myocytes were also spotted in single unconnected cells, suggesting a potential role of Cx43-relevant hemichannels in the regulation of spontaneous Ca^2+^ signaling in normal cardiac myocytes.

Interestingly, a release of ATP or IP_3_ has been found to be responsible for the hemichannel-mediated intercellular Ca^2+^ wave initiation and spreading in non-muscle cells [1], [2], [28], [29]. In this regard, ATP facilitates the Ca^2+^ activities of adjacent unconnected cells by ATP release to the buffer and entrance into these unconnected cells via hemichannels [1], [2], [30], [31]. However, ATP release may not be the main contributor for the hemichannel-related regulation of Ca^2+^ signaling in this study, for suramin, an antagonist of purinergic 2z/2x receptor, did not affect the spontaneous Ca^2+^ transients in the wild type NRVMs, nor did on the potentiated spontaneous Ca^2+^ oscillations in the wt-Cx43 NRVMs (data not shown). In contrast, inhibition of IP_3_R with XeC or 2-APB inhibited the global spontaneous Ca^2+^ signals as well as the gap exchange in NRVMs, whereas blockade of L-type Ca^2+^ channel or RyR, two important effectors of Ca^2+^ signaling, did not show such parallel effects on Ca^2+^ activity and intercellular communication. Importantly, both membrane-permeable IP_3_ and PE, to produce endogenous IP_3_, could completely recover Ca^2+^ oscillations inhibited by heptanol or Gap 27. Moreover, knockdown of the endogenous IP_3_R mimicked the 2-APB effects on NRVMs and, interesting, affected the phosphorylation level of Cx43 (unpublished data in a separate manuscript). Therefore, these data indicate that IP_3_/IP_3_R-associated pathway contributes significantly to Cx43-regulated Ca^2+^ signaling, a proposal is also supported by other studies that demonstrate a direct IP_3_ release mediated by Cx43 [29] and remained inside the cells when gap junction is not formed [36].

In pathological situations, gap junction remodeling characterized by Cx43 lateralization and down-regulation are typical features in failing myocardium [37], [38], in which Ca^2+^ signaling is severely compromised, e.g. lower amplitude and longer duration of Ca^2+^ transient compared with those in normal myocardium [12], [13], [39], [40]. These observations further imply a tight connection between the state/function of connexin and Ca^2+^ signaling in cardiomyocytes. Therefore, the present study demonstrates that in addition to contributing for Ca^2+^ spreading between adjacent cells in normal heart and abnormal Ca^2+^ signal formation in diseased heart, Cx43-associated coupling may also play a role in the regulation of basal intracellular Ca^2+^ activities in normal ventricular myocytes.
